# Dealing with the heterogeneous presentations of freezing of gait: how reliable are the freezing index and heart rate for freezing detection?

**DOI:** 10.1186/s12984-023-01175-y

**Published:** 2023-04-27

**Authors:** Helena Cockx, Jorik Nonnekes, Bastiaan R. Bloem, Richard van Wezel, Ian Cameron, Ying Wang

**Affiliations:** 1 Department of Biophysics, Donders Institute for Brain, Cognition and Behaviour, Radboud University, Heyendaalseweg 135, P.O. Box 9102, 6525AJ Nijmegen, The Netherlands; 2 Department of Rehabilitation, Donders Institute for Brain, Cognition and Behaviour, Radboud University Medical Center, Nijmegen, The Netherlands; 3 Department of Rehabilitation, Sint Maartenskliniek, Nijmegen, The Netherlands; 4 Department of Neurology, Center of Expertise for Parkinson and Movement Disorders, Donders Institute for Brain, Cognition and Behaviour, Radboud University Medical Center, Nijmegen, The Netherlands; 5 Biomedical Signals and Systems Group, Faculty of Electrical Engineering, Mathematics and Computer Science (EEMCS), University of Twente, Enschede, The Netherlands; 6 OnePlanet Research Center, Nijmegen, The Netherlands; 7 ZGT Academy, Ziekenhuisgroep Twente, Almelo, The Netherlands

**Keywords:** Freezing of gait, Movement disorders, Wearable sensors, Heart rate, Accelerometer, Freezing index

## Abstract

**Background:**

Freezing of gait (FOG) is an unpredictable gait arrest that hampers the lives of 40% of people with Parkinson’s disease. Because the symptom is heterogeneous in phenotypical presentation (it can present as trembling/shuffling, or akinesia) and manifests during various circumstances (it can be triggered by e.g. turning, passing doors, and dual-tasking), it is particularly difficult to detect with motion sensors. The freezing index (FI) is one of the most frequently used accelerometer-based methods for FOG detection. However, it might not adequately distinguish FOG from voluntary stops, certainly for the akinetic type of FOG. Interestingly, a previous study showed that heart rate signals could distinguish FOG from stopping and turning movements. This study aimed to investigate for which phenotypes and evoking circumstances the FI and heart rate might provide reliable signals for FOG detection.

**Methods:**

Sixteen people with Parkinson’s disease and daily freezing completed a gait trajectory designed to provoke FOG including turns, narrow passages, starting, and stopping, with and without a cognitive or motor dual-task. We compared the FI and heart rate of 378 FOG events to baseline levels, and to stopping and normal gait events (i.e. turns and narrow passages without FOG) using mixed-effects models. We specifically evaluated the influence of different types of FOG (trembling vs akinesia) and triggering situations (turning vs narrow passages; no dual-task vs cognitive dual-task vs motor dual-task) on both outcome measures.

**Results:**

The FI increased significantly during trembling and akinetic FOG, but increased similarly during stopping and was therefore not significantly different from FOG. In contrast, heart rate change during FOG was for all types and during all triggering situations statistically different from stopping, but not from normal gait events.

**Conclusion:**

When the power in the locomotion band (0.5–3 Hz) decreases, the FI increases and is unable to specify whether a stop is voluntary or involuntary (i.e. trembling or akinetic FOG). In contrast, the heart rate can reveal whether there is the intention to move, thus distinguishing FOG from stopping. We suggest that the combination of a motion sensor and a heart rate monitor may be promising for future FOG detection.

**Supplementary Information:**

The online version contains supplementary material available at 10.1186/s12984-023-01175-y.

## Introduction

Parkinson’s disease is the fastest growing neurological disorder in the world, affecting over 6 million people worldwide. One particularly disturbing symptom of Parkinson’s disease and other parkinsonian disorders is freezing of gait (FOG): a sudden, relatively brief episode of immobility, described by patients as “if the feet are glued to the floor” [1]. This sudden gait arrest affects the lives of at least 2 in 5 people with Parkinson’s disease [2, 3] and it has consequences that reach beyond its effect on locomotion. Research shows an association of FOG severity not only with motor-related dimensions of quality of life like mobility, bodily discomfort, and reduction in activity of daily living, but also with non-motor-related dimensions including emotion, communication, and cognition [4]. Furthermore, clear correlations with disease severity, falls, and hospital admissions result in a high impact on caregivers and the healthcare system [5–7].

There is a great need for methods to reliably measure FOG in the home situation. Given that FOG typically manifests less during hospital visits than at home, it is challenging for clinicians and researchers to track symptom progression [8]. The current gold standard for FOG detection is based on video annotations of two independent trained raters. However, this process is time-consuming, work-intensive, and raises privacy issues when applied in the home setting—potentially holding back the evaluation and development of new treatments for FOG. Several researchers have attempted to detect or predict FOG with the help of motion sensors attached to the body [9–11]. If FOG can be reliably detected by motion sensors alone, this would remove the necessary human labeling element. However, the highly person-specific manifestations of FOG and the risk of overfitting because of small sample sizes are common concerns in these studies.

One of the most frequently applied detection methods is the *freezing index* (FI) developed by Moore et al. [12, 13]. This index is based on the characteristic tremor in the legs during trembling and the absence of frequency components of a normal walking pattern. Based on an accelerometer on the lower limb or trunk, a ratio is calculated of the square of the power in the freezing band (3–8 Hz) over the square of the power in the locomotion band (0.5–3 Hz). When a determined threshold is exceeded, the time window is flagged as a freezing episode.

Despite its simplicity, the performances of the FI varies considerably with a reported sensitivity ranging from 73 to 91% and specificity ranging from 76 to 96% [10]. The heterogeneity of FOG might be at play here [8, 11]. Firstly, FOG presents differently in each person, and in general, three major phenotypes of freezing have been described: (1) *trembling*, characterized by a tremor of the legs at a frequency of 3–8 Hz; (2) *shuffling*, characterized by small steps; and (3) *akinesia*, characterized by a total absence of limb movement (1). Because the FI is specifically designed to detect the trembling type of FOG, it might come up short for the other two types. Secondly, FOG can occur during various circumstances; it can happen during turning, approaching a doorway, initiation of gait, dual-task walking, being under time pressure, etc. [1], but it has never been elucidated whether the FI works equally well under these different circumstances. Therefore, the first aim of this study was to evaluate whether the FI increases significantly under all the different types and triggering situations of FOG.

Another concern of the FI is its vulnerability to falsely classify voluntary stops or ‘normal’ gait events (such as turns without FOG) as FOG [11]. On the one hand, when going from walking to standing, the power in the locomotion band decreases, hence increasing the ratio of the FI. On the other hand, normal gait events might increase the power in the freezing band, hence increasing the ratio of the FI. As a second research aim, we therefore evaluated whether the FI during stopping and during normal gait events (i.e. turns and narrow passages that did not provoke FOG) were statistically different from FOG.

Interestingly, a previous study showed that measuring *heart rate* could also provide signals indicative of FOG that are different from voluntary stopping and turns. Namely, they observed an increase in heart rate before and during FOG, while the heart rate decreased during sudden stops and normal turns [14]. The authors postulated that the increased heart rate might be related to an activation of the autonomic nervous system and increased stress levels. However, only freezing episodes during turning were taken into consideration and the number of FOG episodes was small: about 100 episodes in a total of ten patients. If heart rate indeed increases before and during FOG independently of the type of FOG or its triggering situation, this observation might be promising for future FOG detection or even prediction. Consequently, we evaluated heart rate trends under the same conditions as we evaluated the FI.

Taken together, this study aimed to investigate under which conditions the FI and heart rate might provide reliable signals for FOG detection. In a first analysis, we determined whether the FI and heart rate increased significantly before and during FOG compared to baseline levels. A second analysis tested whether the FI and heart rate during FOG was statistically different from stopping and normal gait events (i.e. turns and narrow passages that did not provoke FOG). For both analyses we specifically evaluated the influence of different types of freezing (i.e., trembling, and akinesia) and evoking situations (i.e. turning, narrow passages; and dual-tasking).

## Methods

### Participants

We recruited 16 participants (11 male, 5 female) diagnosed with idiopathic Parkinson’s disease with self-reported daily freezing. Exclusion criteria were: comorbidities causing severe gait impairments, severe cognitive impairments, or the inability to walk 150 m unaided, possessing a pacemaker, or having a deep brain stimulator.

The severity of motor symptoms was assessed by the Movement Disorder Society’s Unified Parkinson’s Disease Rating Scale section III (UPDRS III) [15]. Furthermore, all participants completed the following questionnaire and cognitive tests: the New Freezing of Gait Questionnaire (NFOGQ) [16], the mini mental state examination (MMSE) [17], and the frontal assessment battery (FAB) [18].

All procedures were conducted according to the principles of the 1964 Declaration of Helsinki and in accordance with the Medical Research Involving Human Subjects Act (WMO). Ethical approval was given by the medical ethics committee Arnhem-Nijmegen (NL60942.091.17). All participants provided written informed consent prior to study inclusion.

### Procedure

To increase the likelihood of FOG occurrence and therefore increasing the power of the study, participants were tested in the OFF medication state following an overnight withdrawal (> 12 h after intake) of anti-Parkinson medication.

**Fig. 1:**
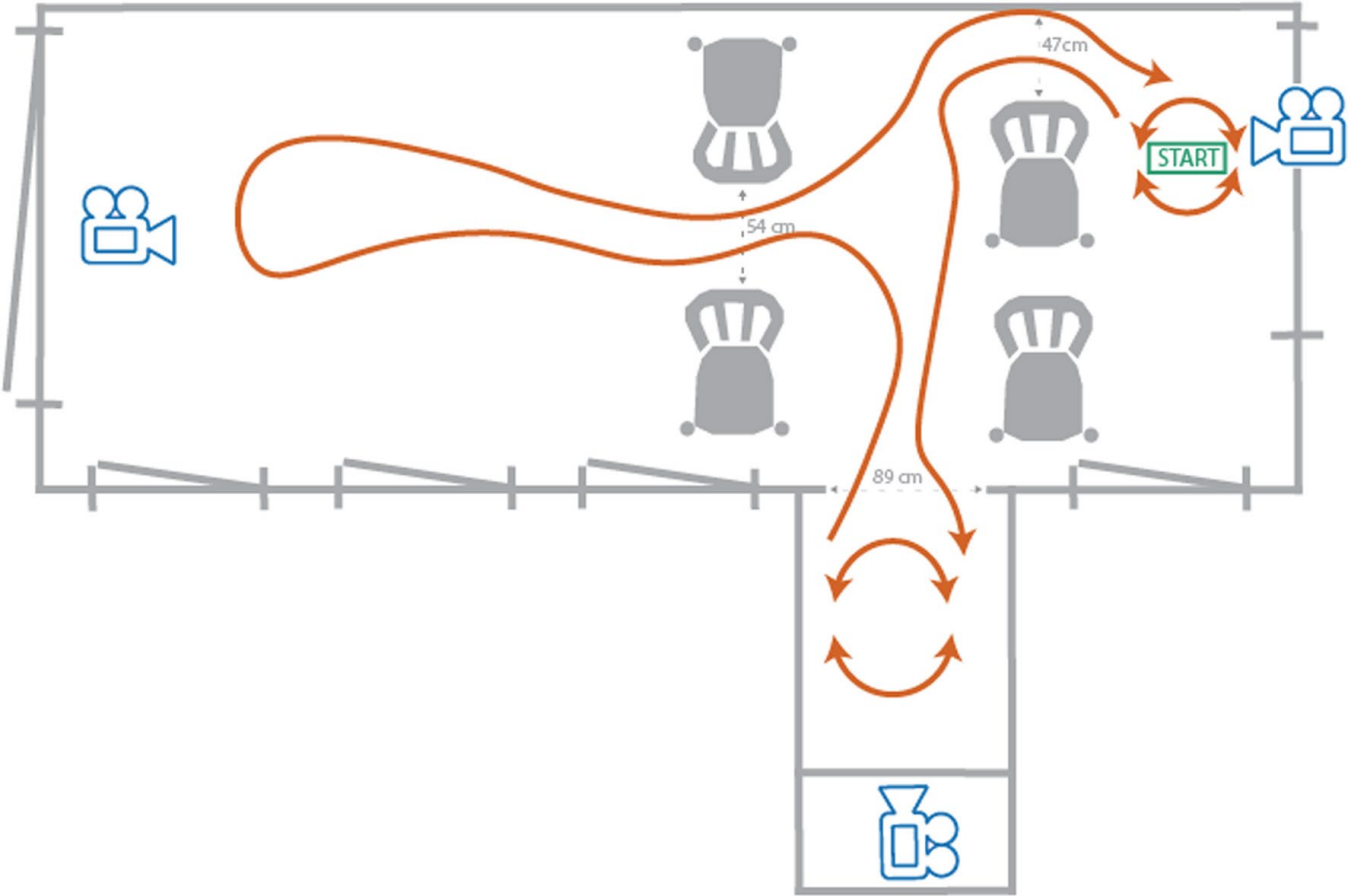
Overview of the performed gait tasks. Each round started with 10 s of standing at the starting point followed by 360 degree turns in alternating directions with and without a cDT (30 s each, in pseudorandomized order). Next, the participants preceded with the gait trajectory as indicated by the orange arrows, passing between the chair and wall (47 cm wide); passing through the doorway (89 cm wide) into the narrow quarter where they completed a 180 and 360 degree turn in alternating directions; passing between the chairs (54 cm wide); walking straight, making a 180 degree turn and walking back between the chairs and the chair and the wall to the starting point where the gait trajectory was started over. The gait trajectory was performed with a cDT, a mDT or noDT for 90 s each, in a pseudorandomized order. In total, at least four of such rounds of approximately 6 min each were completed and in between each round participants could rest as long as needed. All gait tasks were recorded by three video cameras positioned at strategic locations of the lab. (*cDT* cognitive dual-task, *mDT* motor dual-task, *noDT* no dual-task)

A series of gait tasks were designed to maximally trigger FOG during the study visit and to resemble real-life situations. These consisted of 360 degree turns in alternating directions and completion of the gait trajectory shown in Fig. 1, including maneuvering between chairs (i.e. a narrow passage), 180 and 360 degree turns in both directions, stopping, and starting.

The gait tasks were conducted under three different conditions in a pseudo-randomized order: with a cognitive dual-task (cDT), with a motor dual-task (mDT), and without a dual-task (noDT). The cDT consisted of the Adjusted Auditory Stroop Task, which is specifically designed to increase cognitive workload in people with Parkinson’s disease [19]. In short, participants are asked to start or continue walking when hearing a congruent command (i.e., a male voice saying ‘man’ or a female voice saying ‘woman’), and to stand still when hearing an incongruent command (i.e., a male voice saying ‘woman’ or a female voice saying ‘man’). During the mDT, participants carried a tray with an object on it, increasing the demand on the motor system and simultaneously removing vision from the lower limbs [20].

In total, the participants completed four to six rounds of the gait tasks (approximately 6 min each) with each round including the three different DT conditions in a pseudorandomized order. Each round started with 10 s of standing and between each round, the participants could rest as long as needed. During all gait tasks, the participants were not allowed to use a walking aid. They were accompanied by a researcher to prevent them from falling, however, they were required to walk unassisted.

### Materials

A 32-channel portable system (Porti, Twente Medical Systems International B.V., sample frequency of 256 Hz) was used to collect 3-lead electrocardiogram (ECG) signals for heart rate monitoring and 3D accelerometer signals on the ankle and knees for motion activity.

All gait tasks were recorded by three video cameras positioned at different corners of the lab (see Fig. 1) thereby providing an overview of all the participant’s body movements. The videos were annotated for the occurrence of FOG by two independent trained raters in ELAN software (the Language Archive, Nijmegen, the Netherlands) following recent guidelines [21]. A FOG event was considered definite if the annotation of both raters overlapped. Otherwise, the event was in- or excluded after discussion with a third rater. In all cases, we chose the earliest and latest begin and end time from the two raters, respectively, to define the FOG event. This was to prevent the possibility that changes happening before the FOG event can be attributed to annotation differences between the two raters. Furthermore, each time the participants turned, encountered a narrow passage, started, or stopped walking, this was annotated accordingly.

### Data analysis

Raw data were analyzed with MATLAB (R2019a) (Mathworks, Natick, MA) and the open-source Fieldtrip toolbox developed at the Donders Institute for Brain, Cognition and Behaviour [22]. All anonymized data is available on the Donders repository (https://doi.org/10.34973/h1d5-5j25) and analysis scripts are shared on https://github.com/helenacockx/FI-HR_duringFOG. (Note to the editor and reviewers: the URL https://data.donders.ru.nl/login/reviewer-92548469/l5U0Kzu8hnPEyxvfkIs6xDlUpWctA5qGIie5w3FWMwY allows immediate anonymous access for review purposes. Following potential revisions and acceptance, the dataset will be published on the Donders Repository and a persistent DOI will be assigned, which is to be included here).

#### Freezing index (FI)

Because measuring the vertical acceleration of the shin is considered as the most effective for FI calculation and provides the highest signal-to-noise, we only included vertical acceleration of the right shin for calculation of the FI [13, 23–25]. Analysis of the sensor data of the left shin showed similar results. First, the power spectrum for each sample was calculated by using 3-s time windows with Hanning tapers (window centered at the sampling point). The FI was then defined as the squared area under the curve (AUC) of the power in the freezing band (3.5–8 Hz) over the squared AUC of the power in the locomotion band (0.5–3 Hz) as indicated by the following formula [12].

$$FI= \frac{{AUC(power\,freezing\, band \left(3.5-8 Hz\right))}^{2}}{{AUC(power\,locomotion\,band \left(0.5 -3 Hz\right))}^{2}}$$

These values were normalized by multiplying by 100 and taking the natural logarithm as proposed by Moore et al. [12].

#### Heart rate

Prior to analysis, the ECG was examined for heart arrhythmias and the best quality lead was selected. R peaks were detected by the Pan Tompkins algorithm [26, 27]. Singular ectopic beats were automatically identified and replaced by a beat falling exactly between its neighboring beats. Remaining artifacts were selected visually and replaced with *nan* values. To get an indication of the heart rate during rest, we calculated the heart rate and heart rate variability, defined as the coefficient of variation, during the 10 s standing period prior to each round of gait tasks, which was preceded by the optional break.

### Statistics

The FI, and heart rate were averaged over 3-s time windows during FOG (0 to + 3 s after FOG onset), during a preFOG period (− 3 to 0 s before FOG onset), and during baseline (− 6 to − 3 s before FOG onset). Similarly, the same time windows ([− 6 to − 3]; [− 3 to 0]; and [0 to 3] s) were considered for normal gait events and stop events. A normal gait event was defined as a turn or narrow passage without a FOG event within a 6-s margin. A stop event was defined as a voluntary halt, immediately following the command ‘stop’ at the end of each of the DT conditions within the rounds, or following an incongruent command during the cDT condition. Stop events were labeled with the trigger ‘turn’ or ‘narrow passage’, based on its closest gait event. FOG events, stops, or normal gait events preceded by another FOG episode within 6 s were excluded from the analysis.

Mean FI and heart rate values were exported to RStudio (RStudio 1.2.1335; RStudio, Inc., Boston, MA), and analyzed with linear mixed-effects models using the package lme4 [28]. A first model was fitted to compare the FI and the heart rate before and during FOG with baseline levels. The model contained fixed effects for *time* (factors: baseline, preFOG and FOG; treatment contrast coded), FOG *type* (factors: trembling or akinesia; sum contrast coded), FOG *trigger* (factors: turning or narrow passage; sum contrast coded), *DT* condition (factors: cDT, mDT, or noDT; sum contrast coded), and interaction effects of the last four with time. To correct for individual differences in FI thresholds and average heart rate, a random intercept was included for *participant*. Additionally, a random intercept for *trial* was included to baseline correct heart rate levels. It makes no sense to baseline correct the FI because this parameter is usually evaluated based on absolute values rather than relative values, so no random intercept for trial was included for this model. This resulted in the following formulas:

1a $$FI \sim type*time+trigger*time+DT*time+(1|participant)$$

1b $$heart\,rate \sim type*time+ trigger*time+DT*time+\left(1|participant\right)+(1|trial)$$

A second model was fitted to compare FI and heart rate change during FOG to the control conditions ‘normal gait event’ and ‘stopping’. Heart rate change was calculated as the difference in heart rate between the [0 to 3] and [-3 to 0] s time windows. No difference was taken for the FI because this parameter is usually evaluated based on absolute values and not on relative values. The model included fixed effects for *condition* (factors: FOG, normal gait event, and stopping; treatment contrast coded), FOG *type*, FOG *trigger*, *DT* condition, and the interaction effects of the last four with condition. A random intercept was included for *participant*. The formulas were as follows:

2a $$FI \sim type*condition+trigger*condition+DT*condition+\left(1|participant\right)$$

2b $$heart\,rate\,change \sim type*condition+trigger*condition+DT*condition+(1|participant)$$

Assumptions of the models were checked. Fixed effects of all models were tested for statistical significance using Type III F tests with Kenward-Roger adjustment to the degrees of freedom via the car package [29]. Significance level was set at 0.05. Post-hoc analysis for significant main and interaction effects were computed via the emmeans package [30] with Kenward-Roger adjustments to the degrees of freedom and Dunnett’s correction for p-values (in case of significant interaction effects).

## Results

### Participant characteristics

One of the sixteen participants, participant 5, was excluded from analysis because of data loss due to a detached ECG sensor. Another participant, participant 8, although not yet known at the inclusion, eventually was re-diagnosed as having multiple system atrophy (MSA). MSA is a neurodegenerative disorder with similar symptoms as Parkinson’s disease, and people with MSA are often misdiagnosed as having Parkinson’s disease, yet it has a different pathophysiology [31, 32]. We, therefore, decided to analyze participant 8 separately. Table 1 summarizes the participant characteristics of the remaining 14 participants. Median age was 68.0 (interquartile range 59.0–74.8) and median disease duration was 11.0 years (interquartile range 9.0–13.8). Median heart rate during the resting periods was 83.9 bpm (interquartile range 79.6–88.1 bpm); median heart rate variability during rest was 1.6% (interquartile range 1.0–2.3%). The MSA patient had a mean resting heart rate of 77.4 bpm and mean resting heart rate variability of 1.4%.

**Table 1:** Participant characteristics of the 14 included participants

	Median	Interquartile range
Age (years)	68.0	59.0–74.8
Sex (% male)	66.7	
Disease duration (years)	11.0	9.0–13.8
Daily levodopa dosage (mg)	1150.0	1051.0–1350.0
MDS-UPDRS part III	22.0	19.0–24.0
H&Y	2	2–3
MMSE	27.0	26.0–28.5
FAB	16.0	14.3–17.0
NFOGQ	19.0	15.5–22.8
HR rest (bpm)	83.9	79.6–88.1
HRV rest (%)	1.6	1.0–2.3

*MDS-UPDRS part III* Movement Disorders Society Unified Parkinson Disease Rating scale part III, *H&Y* Hoehn and Yahr Staging Scale, *MMSE* mini-mental state examination (range 0–30), *FAB* Frontal Assessment Battery (range 0–18), *NFOGQ* New Freezing of Gait Questionnaire (range 0–28), *HR rest* heart rate during the first 10 s of standing preceding each round*, HRV rest* heart rate variability defined as the coefficient of variation during the first 10 s of standing preceding each round

### FOG annotations

In total, 4683 gait events and 633 freezing episodes were observed in the 14 participants with a high interrater agreement (kappa correlation coefficient 0.83; Spearman correlation for number and total duration of FOG of 0.80 and 0.85 respectively). The median duration of the FOG events was 5.8 s (interquartile range 3.5–11.35 s). For the MSA patient, 37 freezing episodes were observed (kappa correlation coefficient 0.99) with a median duration of 10.9 s.

**Fig. 2:**
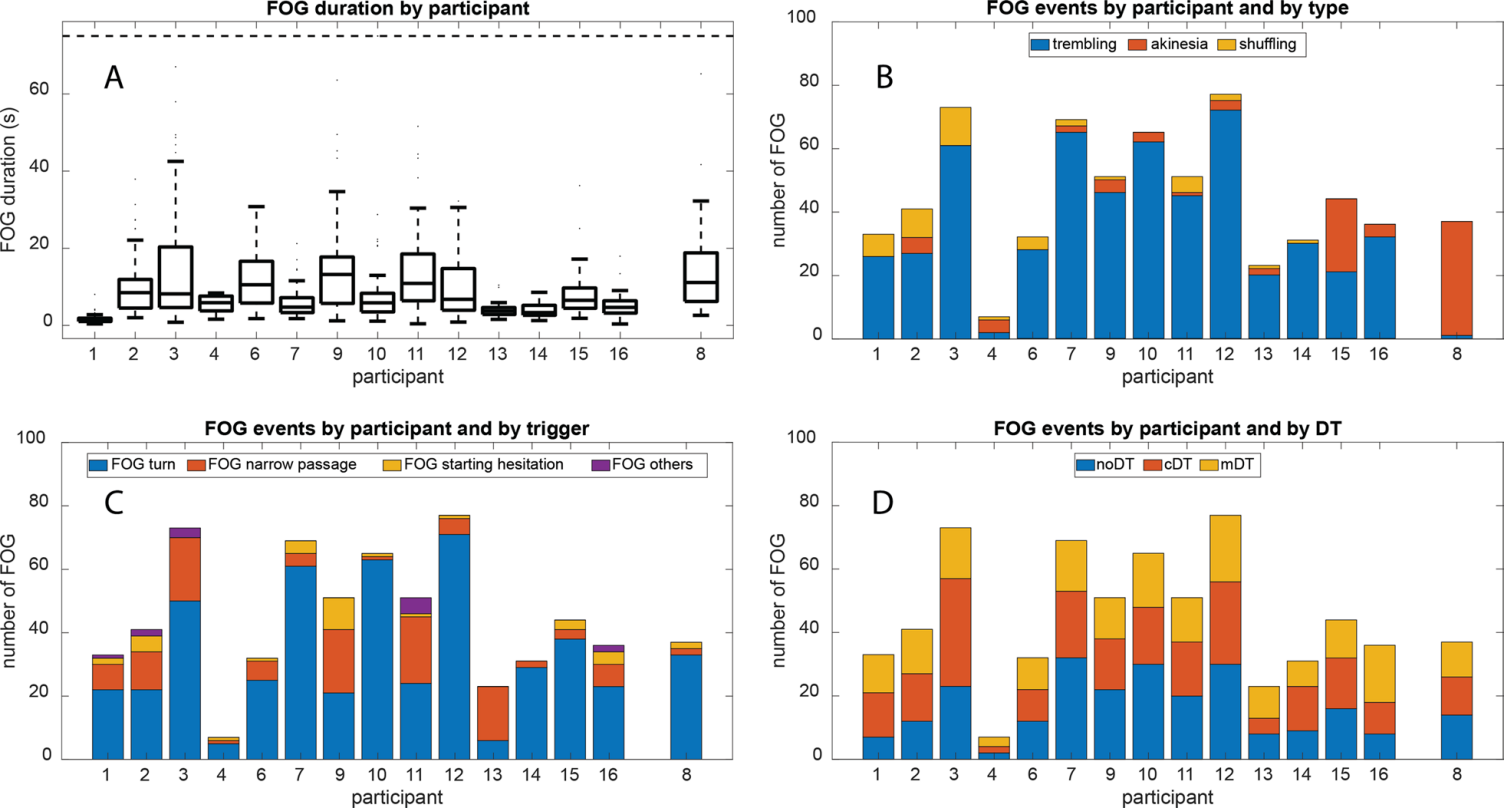
Overview of the annotated FOG events for each participant, including the participant with Multiple System Atrophy (participant 8). **A** boxplots of the FOG durations (s) for each participant. The data is clipped at 75 s (3 events were longer than this upper limit). **B**–**D** Subdivision of the number of annotated FOG events per type (**B**), trigger (**C**), and DT condition (**D)** for each participant. (*FOG* Freezing of Gait; *DT* dual-task)

Figure 2B–D shows the distribution of FOG episodes over the participants for each FOG type, triggering event, and DT condition. 85% of all freezing episodes were annotated as trembling, but was often seen in combination with shuffling (7%), as reflected by a low interrater agreement for both types (in 84% of the shuffling events, the raters disagreed on the FOG type). Because both likely share a similar pathophysiological substrate that is different from akinesia [33, 34], we decided to compare the combination of trembling-shuffling with akinesia (accounting for 8% of the events). Turning was most provocative for FOG (73% of the events), followed by narrow passages (20%) and starting hesitation (5%). The first two, turning and narrow passages, were considered for further analysis. The number of FOG events was comparable over the three DT conditions: 36% of the episodes occurred during noDT, 34% during cDT, and 29% during mDT. After exclusion of trials that were close (< 6 s) to other FOG episodes, 378 FOG events, 351 stops, and 1391 normal gait events were included in the final analysis. For participant 8, 32 FOG events, 29 stops, and 72 normal gait events were retained. Note that this MSA patient mainly displayed FOG of the akinetic type (Fig. 2B)

### Freezing index (FI)

**Fig. 3:**
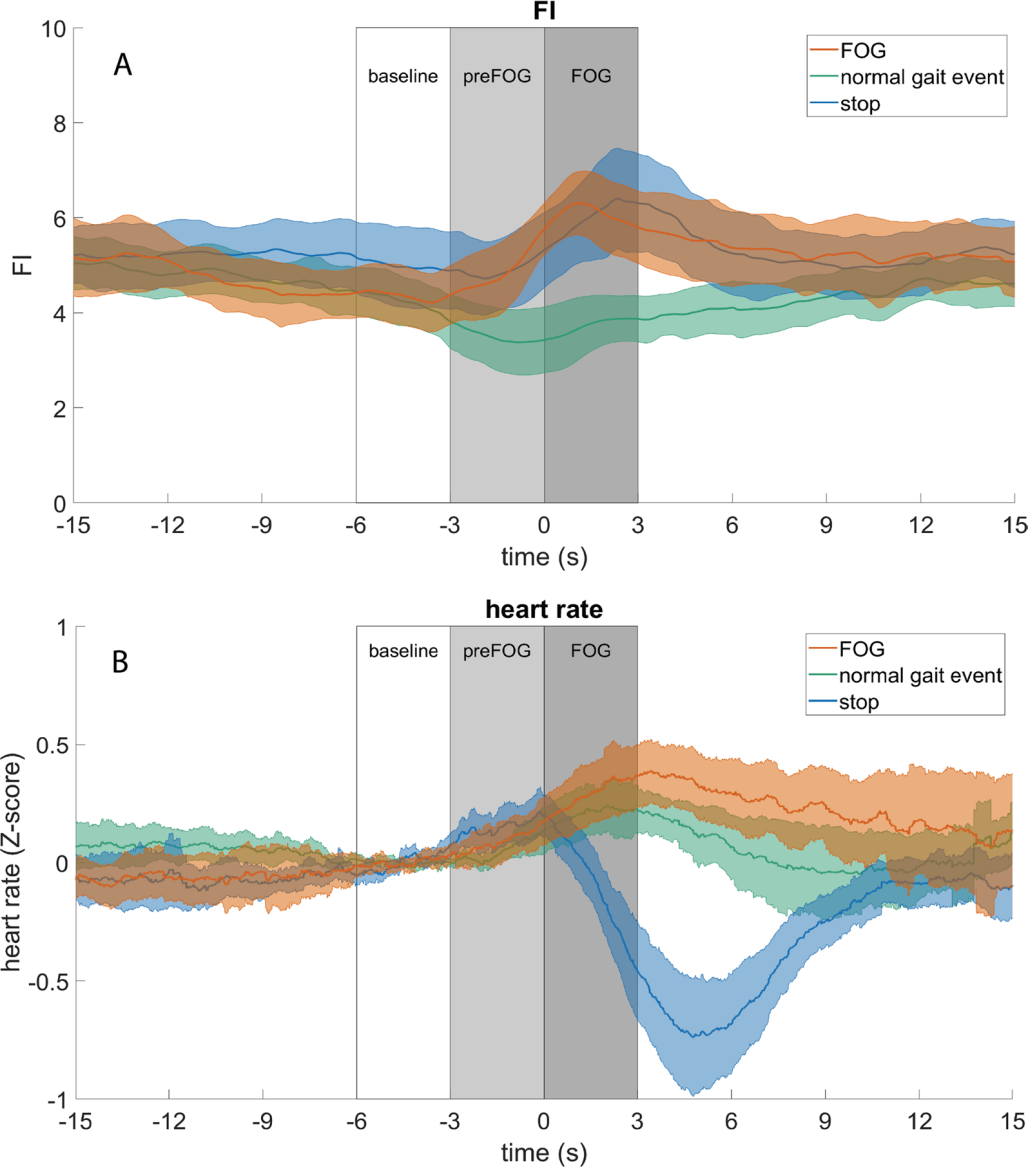
Overall time course of the FI (**A**), and heart rate (**B**) for FOG (orange), normal gait events (green), and, stopping (blue). The lines with the shaded areas represent the mean values with 95% confidence intervals over the 14 included participants (n = 14). The heart rate was z-transformed and baseline corrected (− 6 to − 3 s.) to account for individual variances in baseline heart rate and heart rate variability. No z-transformation was applied to the FI because these values are usually evaluated based on absolute values rather than relative values. The boxes indicate the time intervals over which the variables are averaged when exporting to Rstudio: baseline (− 6 to − 3 s), preFOG (− 3 to 0 s), and FOG (0 to 3 s). The figures were created with MATLAB (R2019a) and the Fieldtrip toolbox. (*FI* Freezing Index, *FOG* Freezing of Gait)

Figure 3A shows the overall time course of the FI during FOG, normal gait events, and stopping. Overall, the FI increased over time during FOG (orange), while it remained at the same level during normal gait events (green). However, the FI also increased during stopping (blue).

**Fig. 4:**
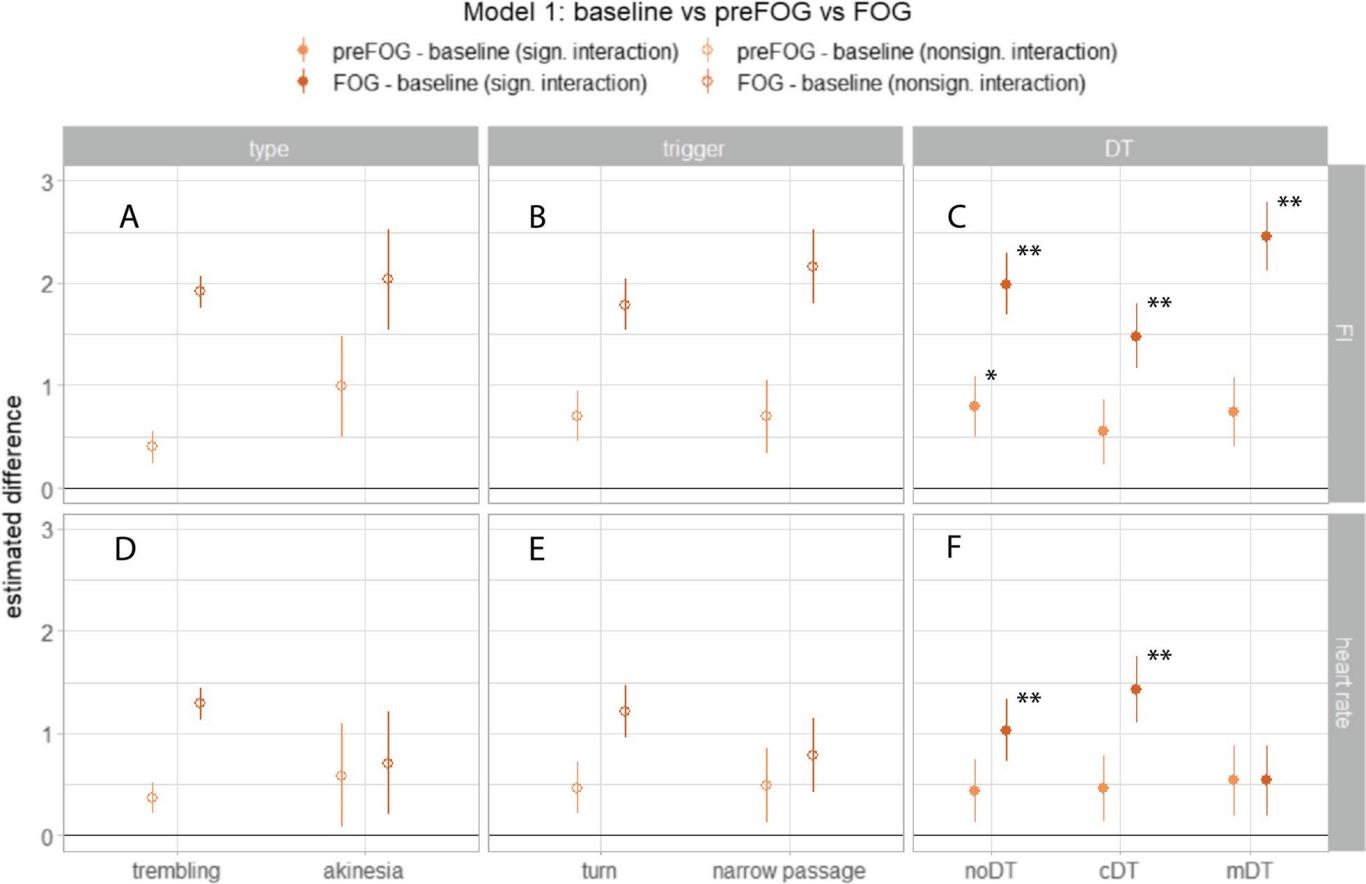
Results of the post-hoc analyses of the linear mixed-model analysis of the first model comparing the FI (**A–C**) (model 1.a), and heart rate (**D–F**) (model 1.b) for the differences between preFOG and baseline (light orange), and FOG and baseline (dark orange). The point ranges indicate the estimated differences with standard errors of the post-hoc analyses for each FOG type (first column), FOG trigger (second column), and DT condition (third column), but the symbols are only filled when significant interaction effects were found for this factor by time. This means that the panels with the hollow symbols followed the main effects of Table 2. Results of the post-hoc analysis that were significant after p-value correction are indicated with an asterisk (*, < 0.05; **, < 0.005). Figures were created with the ggplot2 package in Rstudio. (*FI* Freezing Index; *FOG* Freezing of Gait; *noDT* no dual-task; *cDT* cognitive dual-task; *mDT* motor dual-task)

The first model (model 1.a), evaluating the increase of the FI during preFOG and FOG compared to baseline levels, showed that the FI increased significantly over time (main effect of time: FI_(time)_: p < 0.001) (Table 2). Figure 4A–C shows the results of the post-hoc analyses of this model. We found a significant interaction effect for DT (FI_(time*DT)_: p = 0.015), with a higher increase of the FI during noDT than during the cDT and even higher during the mDT, though in all cases significantly different from baseline (p < 0.001). The significant main effects for trigger (p = 0.015), and for DT (p < 0.001) indicate that the FI was different at baseline for these conditions. Overall, these results show that the FI performed as expected (i.e., significant increase compared to baseline) in all tested circumstances and for all types of FOG, including akinesia.

**Table 2:** Results of the type III F test (with Kenward-Roger adjustment for degrees of freedom) for the fixed effects of the mixed models

	Model 1.a: FI ~ type*time + trigger*time + DT*time + (1|participant)				Model 1.b: heart rate ~ type*time + trigger*time + DT*time + (1│participant) + (1|trial)			
	F	Df	Df.res	p value	F	Df	Df.res	p value
(Intercept)	136.63	1	30	< 0.001*	1248.09	1	14	< 0.001*
Time	24.18	2	1106	< 0.001*	5.64	2	746	0.004*
Trigger	5.90	1	1112	0.015*	8.45	1	439	0.004*
Type	0.00	1	1114	0.992	3.12	1	430	0.078
DT	12.12	2	1106	< 0.001*	7.96	2	447	< 0.001*
Time*type	0.83	2	1106	0.435	1.40	2	746	0.246
Time*trigger	1.03	2	1106	0.356	1.42	2	746	0.241
Time*DT	3.09	4	1106	0.015*	3.11	4	746	0.015*

	Model 2.a: FI ~ type*condition + trigger*condition + DT*condition + (1│participant)				Model 2.b: heart rate change ~ type*condition + trigger*condition + DT*condition + (1|participant)			
	F	Df	Df.res	p value	F	Df	Df.res	p value
(Intercept)	347.30	1	28	< 0.001*	2.65	1	464	0.104
Condition	91.30	2	2095	< 0.001*	54.07	2	2091	< 0.001*
Trigger	0.60	1	2096	0.439	3.23	1	2096	0.073
DT	0.64	2	2094	0.528	5.74	2	2103	0.003*
Type	0.02	1	2097	0.888	2.20	1	2062	0.138
Condition*trigger	47.47	2	2094	< 0.001*	3.83	2	2101	0.022*
Condition*DT	2.06	4	2093	0.084	3.28	4	2101	0.011*
Condition*type	0.58	2	2095	0.558	2.28	2	2092	0.102

*p < 0.05; *F* F values, *Df* degrees of freedom, *Df.res* residual degrees of freedom, *FI* freezing index, *DT* dual-task

**Fig. 5:**
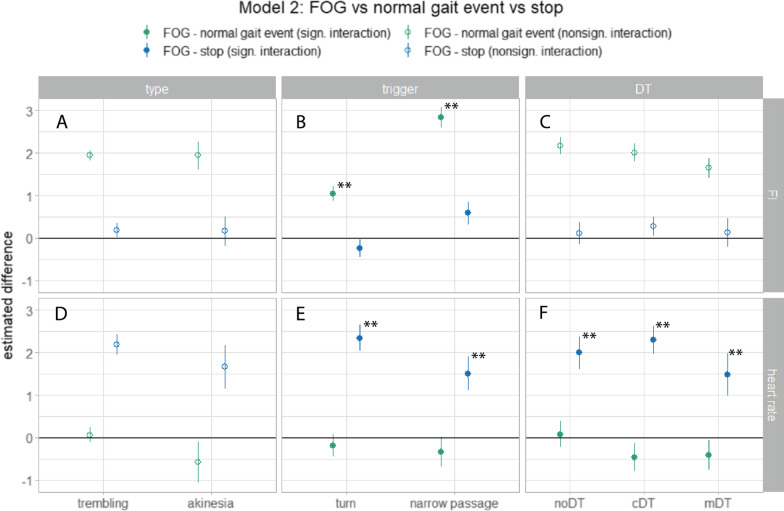
Results of the post-hoc analyses of the linear mixed-model analysis of the second model comparing the FI (**A–C**) (model 2.a), and heart rate change (**D**–**F**) (model 2.b) for the differences between FOG and a normal gait event (green), and FOG and stopping (blue). The point ranges indicate the estimated differences with standard errors of the post-hoc analyses for each FOG type (first column), FOG trigger (second column), and DT condition (third column), but the symbols are only filled when a significant interaction effect of this factor was found with condition (i.e. FOG—normal gait event or FOG—stop). This means that the panel with the hollow symbols followed the main effects of Table 2. Results of the post-hoc analysis that were significant after p-value correction are indicated with an asterisk (*, < 0.05; **, < 0.005). Figures were created with the ggplot2 package in Rstudio. (*FI* Freezing Index, *FOG* Freezing of Gait, *noDT* no dual-task, *cDT* cognitive dual-task, *mDT* motor dual-task)

Figure 5A–C shows the results of the second model (model 2.a) that compared the FI during FOG, normal gait events, and stops under all conditions. For the ideal FOG detector, the green (FOG—normal gait event), and blue (FOG—stopping) point ranges would appear above the zero threshold line. A significant main effect for condition (p < 0.001) (Table 2) signaled that FI performed better to distinguish FOG from normal gait events (post hoc FI_(FOG-normal gait event)_: p < 0.001) than to distinguish FOG from stopping (post hoc FI_(FOG-stop)_: p = 0.592).

A significant interaction effect was found between condition and trigger (FI_(condition*trigger)_: p < 0.001, indicating that the relation of the FI between FOG and a normal gait event was different between turning and a narrow passage (Fig. 5B). In a narrow passage, the FI was higher during an event with FOG than without FOG (post-hoc FI_(FOG-normal gait event)_: B = 2.84, p < 0.001); but for turning, the relationship became smaller (post-hoc FI_(FOG-normal gait event)_: B = 1.04, p < 0.001).

Taken together, these results show that the FI performs well to differentiate FOG from normal gait events (although these differences were smaller when comparing FOG turns with normal turns), but not to differentiate FOG from stopping.

### Heart rate

Figure 3B shows that overall, heart rate increased during FOG compared to baseline levels, while a clear drop in heart rate was seen during stopping. The heart rate increased also during normal gait events.

Indeed, the first model (model 1.b) showed a significant main effect of time (HR_(time)_: p = 0.004), with post-hoc analysis revealing a significant increase in heart rate during FOG compared to baseline (HR_(FOG-baseline)_: B = 1.00, p < 0.001), but not during preFOG (HR_(preFOG-baseline)_: B = 0.48, p = 0.146) (Table 2). The increase in heart rate was dependent on DT condition (HR_(time*DT)_: p = 0.015), but not on type (HR_(time*type)_: p = 0.246), or on the triggering event (HR_(time*trigger)_: p = 0.241). Figure 4F shows indeed that the heart rate increased significantly during FOG for the noDT and the cDT condition (post-hoc HR_(FOG-baseline)_: B = 1.03, p = 0.002 and HR_(FOG-baseline)_: B = 1.43, p < 0.001), but not for the mDT condition (HR_(FOG-baseline)_: B = 0.54, p = 0.21).

Of less interest, a significant main effect of the first model was found for trigger (HR_(trigger)_: p = 0.004) and DT (HR_(DT)_: p < 0.001), meaning that the heart rate levels for these conditions differed at baseline (Table 2). Namely, heart rate was higher during baseline levels of narrow passages than during turning (post-hoc HR_(narrow passage—turn)_: B = 1.51, p = 0.005), and heart rate during baseline levels of mDT was higher than during cDT (HR_(mDT-cDT)_: B = 1.94, p < 0.001).

Overall, these results confirm that heart rate increases during FOG and this was independent of the type of freezing. Besides, the heart rate during the mDT condition had significantly higher baseline levels and did not increase significantly during FOG.

The second model (model 2.b) investigated the differences in heart rate changes during FOG with stopping and normal gait events (Fig. 5D–F)*.* A significant main effect of condition (p < 0.001) indicated that the heart rate change during FOG was different when compared to stopping than when compared to normal gait events. Namely, the heart rate change during FOG was significantly different from stopping (post hoc HR_(FOG-stop)_: B = 1.93, p < 0.001) while it was similar to normal gait events (post hoc HR_(FOG-normal gait event)_: B = -0.26, p = 0.536).

A significant interaction effect between condition and trigger (p = 0.022) was observed with a slightly smaller difference in heart rate change between FOG and stopping during a narrow passage than during a turn (Fig. 5E). Moreover, a significant interaction effect was observed between condition and DT with a slightly smaller difference in heart rate change between FOG and normal gait event for the noDT condition than the other two conditions (Fig. 5F). In addition, a significant main effect of the second model was seen for DT (HR_(DT)_: p = 0.003) indicating differences in heart rate change for these factors during the FOG condition (Table 2). More specifically, heart rate change was greater during cDT than noDT (post-hoc HR_(cDT-noDT)_: B = 0.46, p = 0.001).

In conclusion, heart rate change was significantly different between FOG and stopping of any type and triggering situation, but not between FOG and a normal gait event.

### Participant with MSA

We present the overall time courses of the FI and heart rate for participant 8 separately (Additional file 1: Fig. S1) as this person was re-diagnosed as having multiple system atrophy (MSA) instead of idiopathic Parkinson’s disease. Note that this participant mainly had FOG of the akinetic type (Fig. 2B). The FI slightly decreased during FOG, while it increased during stopping. The heart rate neither increased nor decreased during FOG, while it decreased during voluntary stopping.

## Discussion

We set out to characterize the FI and heart rate during FOG in different situations to evaluate under which conditions these features might provide reliable signals for FOG detection. Overall, the FI worked well to distinguish FOG from normal walking (significant increase compared to baseline levels) or from normal gait events, but had difficulties to distinguish FOG from stopping or to detect FOG during turning. In contrast, the heart rate change could clearly differentiate between stopping and FOG of all types or during any triggering situation, but did not show significant differences from normal gait events.

### The freezing index (FI)

For this study, we specifically compared FOG to stopping and to FOG-provoking events without FOG (i.e. normal gait events). We see this approach as a major strength of our study, as previous work usually ignored these types of events, like voluntary stops or normal turns, neglecting the specific investigations for potential false positive events [13, 35]; or only compared the overall FOG severity calculated by the algorithm to the FOG severity rated on video [36].

We found an increase in the FI for both trembling and akinetic FOG, however, both types were indiscernible from voluntary stops as the FI also increased during this event. The increase in the FI during stopping can be explained as follows: when no movements are performed, both the power in the freezing band and locomotion band take very small values and only contain remaining sensor noise [24]. These low values, certainly of the locomotion band, can give rise to unstable ratios, and results during stopping in an increase of the FI. This faulty increase of the FI durint stops might be even more problematic than previously reported by Moore and colleagues [12] who observed only a 20% false positive rate during volitional standing. A similar mechanism might be at play during akinetic FOG, for which we indeed observed a decrease in both the freezing and locomotion band, resulting in a net increase in the FI. For the participant with MSA, who mainly displayed akinetic FOG, the decrease in both bands took slightly different proportions, resulting in a net *decrease* in the FI. So the FOG episodes of this participant were not being flagged as a FOG at all. We overall conclude that the FI may result in unreliable ratios when the power in the locomotion band is low, e.g. during stopping or akinetic FOG.

Some researchers have proposed a solution to deal with the rising FI during normal stops, by introducing an extra threshold: only when the total power (0.5–8 Hz) exceeds a certain total power threshold, is the FI calculated [23, 24]. We argue, however, that the performance of this modified version of the FI (mFI) is unsatisfactory. Additional file 1: Fig. S2 shows the overall time course of mFI during FOG, normal gait events, and stopping. Indeed, the mFI increased less during stops than the FI, but was still not significantly different from FOG. Moreover, the difference in the mFI between FOG and normal gait events became smaller. We, therefore, argue that the extra total power threshold marginally improved the distinction between FOG and stopping, but at the cost of differentiating between FOG and normal gait events.

Regarding the different triggering situations, the results of our mixed model analysis showed that the difference between turns with and without FOG was smaller compared to the other situations. Previous studies likewise reported a large number of false positive ratings (67 out of 202 accelerometer-detected FOG episodes) when using the FI to detect FOG during turning [35] or reported lower performances during turn-like behavior [37]. These results can be explained by a similar increase in the FI during turns with and without FOG. Indeed, we observed a higher increase in the freezing band than in the motor band during normal turning, resulting in a net increase in the FI, similarly like observed in one of our previous studies [38]. For this study, we particularly asked participants to turn on the spot with small steps instead of big steps or a pirouette, thereby probably increasing the power in the 3.5–8 Hz band, hence explaining the increase in the FI. These instructions were given to increase the likelihood of triggering FOG, but represent, nevertheless, a turning behavior with high risks to provoke FOG. This observation, therefore, underlines the importance of not only comparing FOG events to walking or standing, but also to its triggering events without FOG (e.g., normal turning). Likewise, researchers should specifically consider FOG events triggered by different conditions when developing FOG detection algorithms, to ensure detection performance in daily life.

### Heart rate

Consistent with previous studies, we found a significant increase in heart rate during FOG [14, 39, 40]. This increase in heart rate was present for all types and triggering situations, except for the mDT condition. More importantly, the significant difference with the clear heart rate drop during stopping was clearly persistent for all the conditions, including the mDT condition (the blue point ranges in Fig. 5D–F are always far above zero). This can also be explained biologically*:* autonomic adjustments of the heart rate during exercise, which are both centrally and locally regulated, are dependent on a person’s perceived effort, independently of whether the attempt to move is successful or not [41, 42]. For example, if stuck in deep snow, you make great efforts to escape, which increases your heart rate, but your efforts might be fruitless. This analogy also makes sense for akinesia, during which people’s feet almost literally are glued to the floor. Following on from this, it seems logical that heart rate decreases during voluntary stopping, while it increases during the efforts to move during FOG. Previous studies attribute the increase in heart rate during FOG to elevated stress levels [14, 40]. Our findings do not contradict this hypothesis, but we note that not only stress may play a role, but that the increased heart rate can also be a normal physiological response to exertion.

It remains unclear, however, why heart rate did not increase during FOG for the mDT condition. We speculate that saturation effects might have played a role. The mDT condition displayed higher heart rate levels during baseline, which could be explained by the imposed increase in mental workload of carrying the tray [43]. Autonomic cardiovascular dysfunction is prevalent in Parkinson’s disease, resulting in lower heart rate variability, thereby possibly preventing a further increase of the already high baseline levels [44]. Because the cDT included an episodic type of DT, namely starting or stopping based on episodic auditory stimuli, this condition had lower heart rate baseline levels and therefore a significant heart rate increase during FOG. Nevertheless, although heart rate did not increase during the mDT, the difference in heart rate change with stopping was still significant.

The increase in heart rate during FOG was less clear for the patient with multiple system atrophy (MSA). FOG is estimated to be present in 40–75% of MSA patients [45, 46], but the pathophysiology of FOG in MSA might be distinct from Parkinson’s disease [47]. Furthermore, autonomic dysfunction is prevalent in MSA [32], potentially blunting cardiovascular reflexes. Nevertheless, more research is needed to investigate the differences in neural pathways and cardiovascular responses during FOG in MSA and other parkinsonian syndromes.

While the difference in heart rate change between FOG and stopping is strong, no clear differences in heart rate change between FOG and normal gait events are observed, for none of the conditions (green point ranges in Fig. 5D–F are close to zero). This is in contrast with findings by Maidan and colleagues [14] where heart rate decreased during a normal turn, but similar to findings by our research team [38]. This disagreement with the first study may be due to a difference in effort during turning, for example, because the participants in our study turned with shorter and faster steps. However, this does not explain the increased heart rate when encountering a narrow passage. Alternatively, we can explain this by a conditioning mechanism; as chances of freezing grow when turning or approaching a narrow passage, the perceived exertion or stress levels rise, causing heart rate to increase.

### Recommendations

The main purpose of this study was to evaluate the use of motion sensors and heart rate monitors for future FOG detection algorithms. Although only looking at group effects in the data rather than trials separately, the FI seemed to perform reasonably well to distinguish FOG from walking or normal gait events. However, we also showed it might confuse stopping with FOG, both for trembling and akinetic types. Because FOG is particularly variable in presentation, it is challenging to acquire a one-size-fits-all algorithm based on motion sensors. Of course, person-specific models can be trained, but in practice, this means that for each individual a new annotated dataset needs to be generated [10].

**Fig. 6:**
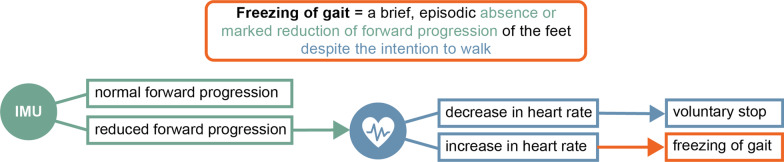
Graphical representation of how a hypothetical new FOG detection method would work by combining a motion sensor (IMU) and a heart rate monitor. If a motion sensor would first measure a reduction in forward progression (e.g. IMU close to the center of gravity like a phone in the back pocket or a sensor measuring the speed of the wheels of a walker), a heart rate monitor (e.g. smartwatch) could subsequently define whether this reduction in forward progression was voluntary (stop) when accompanied by a decrease in heart rate, or involuntary (FOG) when not accompanied by a decrease in heart rate. (*IMU* Inertial Measurement Unit; *FOG* freezing of gait)

Instead of trying to characterize all possible FOG patterns using leg-mounted motion sensors, we can also focus on what FOG has in common (Fig. 6). During the 2010 FOG workshop, freezing of gait was unified under the following definition: “a brief, episodic absence or marked reduction of forward progression of the feet despite the intention to walk” [48, 49]. It should be relatively simple to measure a reduction in forward progression, for example with an inertial measurement unit (IMU), close to the center of gravity (e.g. a phone in the back pocket) [50], by measuring the speed of the wheels of a walker, by measuring variation in gait cadence [23], or by measuring the cross-correlation between the left and right foot angular velocity as proposed by a recently published study [36]. We hereby recommend the use of a simple metric measuring forward progression instead of using the FI, which may result in unreliable ratios when the power in the locomotion band is low. The second part of the definition—‘despite the intention to walk’—is crucial for differentiating FOG from voluntary stopping. As explained previously, the heart rate can reflect someone’s effort to move independent of its success, hence opening the possibility to distinguish voluntary from involuntary stops. The results of this study clearly demonstrated that heart rate indeed can make this distinction for any type and situation of FOG. Therefore, we suggest that the combination of a motion sensor (measuring forward progression), and a heart rate monitor (measuring the intention to move), could improve FOG detection algorithms by better distinguishing FOG from stopping. However, future work using a multimodal approach, for example as proposed in one of our previous studies [38], should verify whether such a system would indeed lead to improved FOG detection metrics.

While heart rate is easy to measure and smartwatches that collect heart rate data are readily available, certain aspects should be taken into consideration. Firstly, heart rate monitors should be precise enough to pick up small differences. The difference in heart rate change between FOG and stopping counted only 1.78 bpm on average, so devices should be able to detect differences of approximately 1–2 bpm. Secondly, autonomic dysfunction is prevalent in Parkinson’s disease and some patients might take beta-adrenergic blocking agents for tremor reduction, thereby blunting possible cardiovascular responses [44]. As a consequence, variances in heart rate might become so small that even very precise monitors are not able to detect relevant changes. Thirdly, other changes in the physical, emotional, and cognitive state may modulate heart rate. Therefore, the effect of such states during standing should be investigated and compared to FOG events. Lastly, although this concept sounds promising for akinesia detection, more data is needed to confirm this hypothesis.

### Limitations

We acknowledge a couple of limitations. Firstly, the goal of this study was to look at group-level average data in the FI and the heart rate to investigate future directions for FOG detection algorithms. Therefore, we cannot draw conclusions on the performance of the FI and heart rate on a single trial level. Nevertheless, by analyzing the data with linear mixed models, single trials were taken into consideration while being corrected for differences between participants. Secondly, the FI and heart rate were averaged over 3-s time windows potentially not capturing the very short FOG episodes. However, over 80% of the FOG episodes were longer than 3 s and excluding the shorter trials did not influence the data substantially. Nevertheless, future research should conclude whether measuring heart rate would still be useful for detection of short FOG episodes. Thirdly, the gait trajectory included many tasks that succeeded each other rapidly. Although we excluded trials that were preceded by a FOG within 6 s, carry-over effects cannot entirely be eliminated. Lastly, the amount of data on akinetic FOG was limited. The results on akinesia should therefore be interpreted with care. Nevertheless, the difference in heart rate change between akinesia and stopping was consistent. We, therefore, believe that the heart rate remains a promising feature for akinesia detection, certainly because it remains a challenge to distinguish it from voluntary stops with the use of motion sensors, or even by looking at videos.

## Conclusion

We clearly showed that the FI can differentiate well between FOG and normal gait events, but has issues to distinguish FOG from stopping, and can produce false-positives during other gait events like normal turns. Subsequently, we confirmed that heart rate increases during different types of FOG episodes. We suggest that the significant difference in heart rate change between any type or condition of FOG and stopping may help to improve FOG detection algorithms based on motion sensors measuring forward body progression. This concept might be particularly promising for akinesia detection, although more data is required to confirm this finding.

## Supplementary Information

Additional file 1: Figure S1. Overall time course of the FI and heart rate for participant 8. Figure S2. Overall time course of the modified FI.

## Data Availability

The anonymized dataset supporting the conclusions of this article is available in the Donders Repository in BIDS-like format (https://bids.neuroimaging.io/): https://doi.org/10.34973/h1d5-5j25. All analysis code of this manuscript can be found on https://github.com/helenacockx/FI-HR_duringFOG.

## Abbreviations

AUC: Area under the curve
cDT: Cognitive dual-task
DT: Dual-task
ECG: Electrocardiography
FAB: Frontal Assessment Battery
FI: Freezing index
FOG: Freezing of gait
HR: Heart rate
HRV: Heart rate variability
MDS-UPDRS: Movement Disorders Society Unified Parkinson Disease scale
mDT: Motor dual-task
mFI: Modified freezing index
MMSE: Mini-mental state examination
MSA: Multiple System Atrophy
NFOGQ: New freezing of gait questionnaire
noDT: No dual-task
