# Correction: Dealing with the heterogeneous presentations of freezing of gait: how reliable are the freezing index and heart rate for freezing detection?

**DOI:** 10.1186/s12984-023-01196-7

**Published:** 2023-06-10

**Authors:** Helena Cockx, Jorik Nonnekes, Bastiaan R. Bloem, Richard van Wezel, Ian Cameron, Ying Wang

**Affiliations:** 1 Department of Biophysics, Donders Institute for Brain, Cognition and Behaviour, Radboud University, Heyendaalseweg 135, P.O. Box 9102, 6525AJ Nijmegen, The Netherlands; 2 Department of Rehabilitation, DondersInstitute for Brain, Cognition and Behaviour, Radboud University Medical Center, Nijmegen, The Netherlands; 3 Department of Rehabilitation, Sint Maartenskliniek, Nijmegen, The Netherlands; 4 Department of Neurology, Center of Expertise for Parkinson and Movement Disorders, Donders Institute for Brain, Cognition and Behaviour, Radboud University Medical Center, Nijmegen, The Netherlands; 5 Biomedical Signals and Systems Group, Faculty of Electrical Engineering, Mathematics and Computer Science (EEMCS), University of Twente, Enschede, The Netherlands; 6 OnePlanet Research Center, Nijmegen, The Netherlands; 7 ZGT Academy, ZiekenhuisgroepTwente, Almelo, The Netherlands

**Correction: Journal of NeuroEngineering and Rehabilitation (2023) 20:53** **https://doi.org/10.1186/s12984-023-01175-y**

Following publication of the original article [1], the middle name of the author Bastiaan R. Bloem has been updated in the author group.

The original article has been corrected.
