# Supplementary material for: Dealing with the heterogeneous presentations of freezing of gait: how reliable are the freezing index and heart rate for freezing detection?
Source: J Neuroeng Rehabil. 2023 Apr 27;20:53. doi: 10.1186/s12984-023-01175-y (PMC10134593; doi:10.1186/s12984-023-01175-y)
Supplement: Supplementary file 1 — Additional file 1: Figure S1. Overall time course of the FI and heart rate for participant 8. Figure S2. Overall time course of the modified FI. [file 12984_2023_1175_MOESM1_ESM.pdf]

Additional file 1: Fig. S1: Overall time course of the FI and heart rate for participant 8

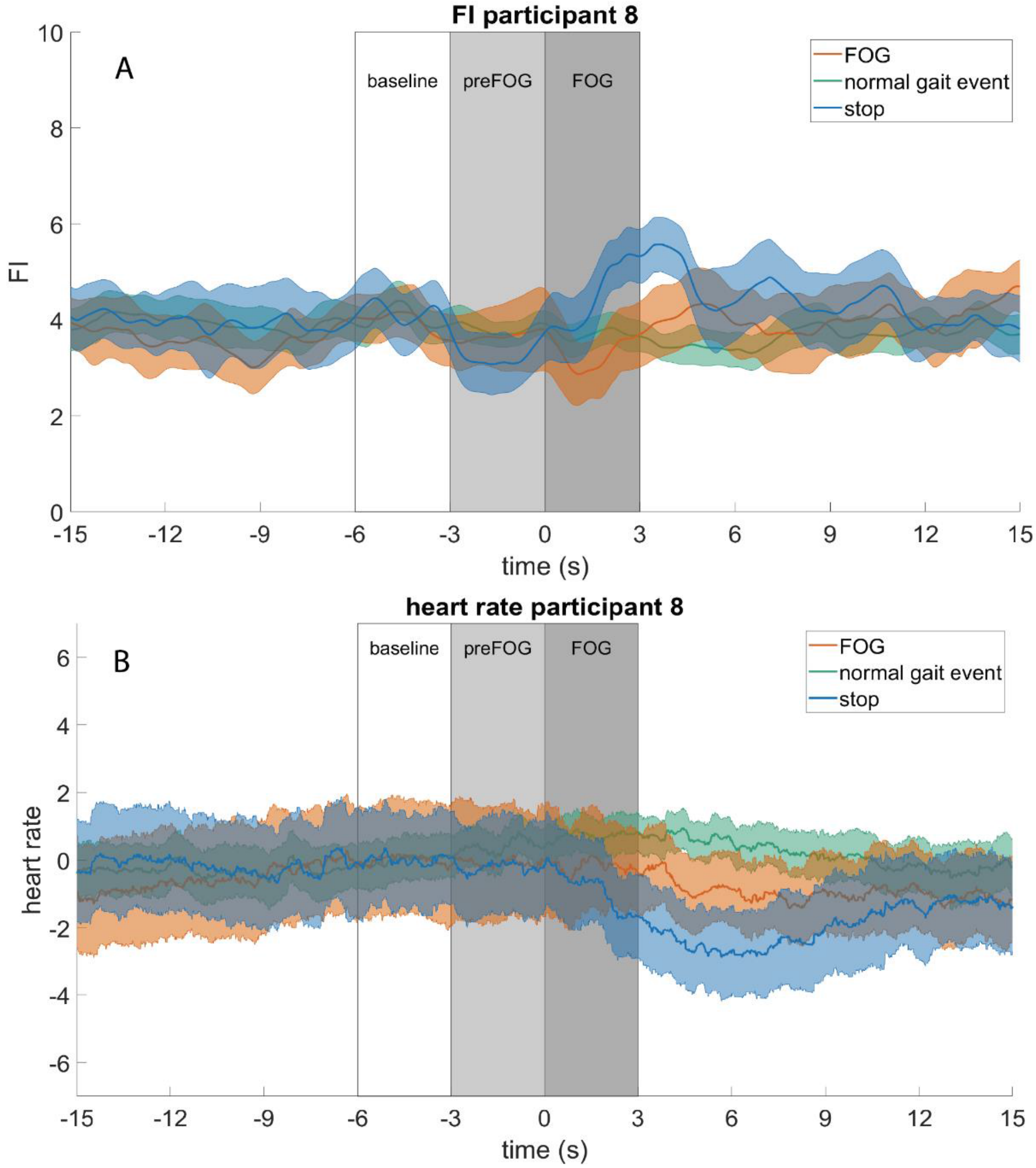

Additional file 1: Fig. S1: Overall time course of the Freezing Index (FI) (A), and baseline corrected (-6 to -3 s) heart rate (B) for participant 8 (MSA patient) during FOG (orange), normal gait events (green), and stopping (blue). The lines with the shaded areas represent the mean values with confidence intervals over the included events. (FI = Freezing Index; MSA = Multiple System Atrophy; FOG = Freezing of Gait)

Additional file 1: Fig. S2 : overall time course of the modified FI

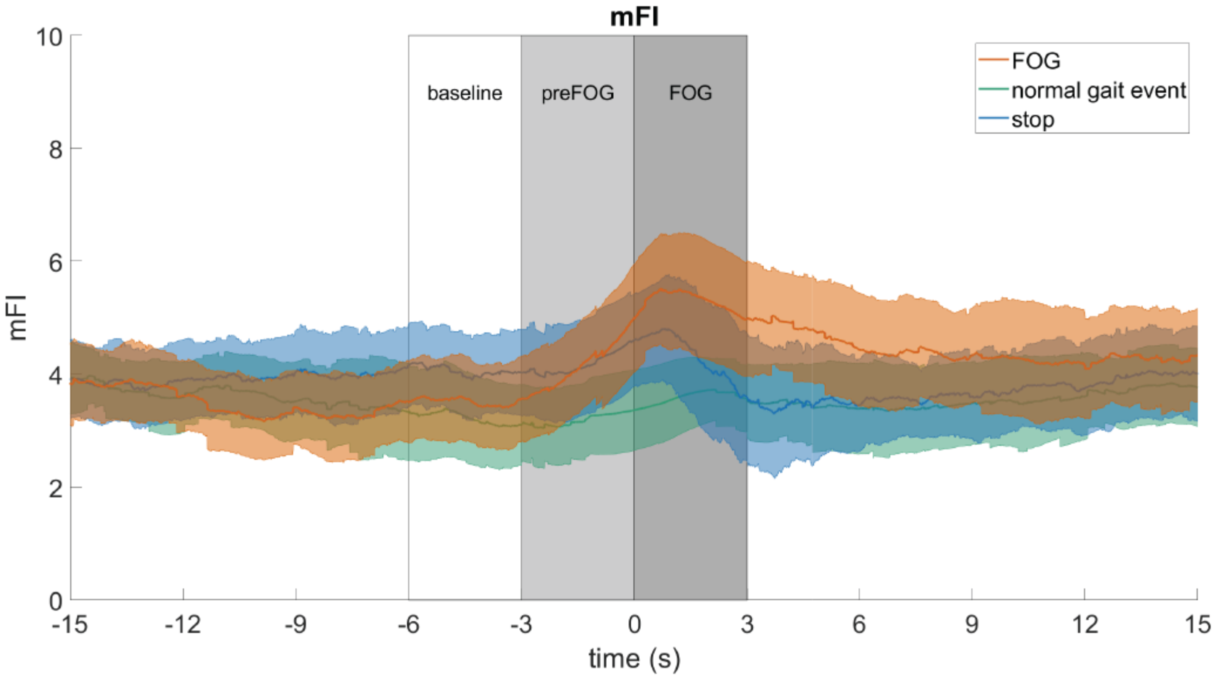

Additional file 1: Fig. S2: Overall time course of the “modified” FI (mFI) for FOG (orange), normal gait events (green), and, stopping (blue). The lines with the shaded areas represent the mean values with confidence intervals over the 14 included participants (n=14). To calculate the mFI, we defined a total power threshold as the mean plus one standard deviation of the total power (0.5-8 Hz) during the 10 s of standing preceding each round(1). Only when the total power (0.5-8 Hz) exceeded this threshold, the FI was calculated, otherwise the FI was set at zero. The figures were created with MATLAB (R2019a) and the Fieldtrip toolbox. (*FI* = Freezing Index; *mFI* = modified Freezing Index; *FOG* = Freezing of Gait)
